# Mechanism and Potential of Extracellular Vesicles Derived From Mesenchymal Stem Cells for the Treatment of Infectious Diseases

**DOI:** 10.3389/fmicb.2021.761338

**Published:** 2021-10-26

**Authors:** Jingyi You, Zhou Fu, Lin Zou

**Affiliations:** ^1^Department of Respiratory Children’s Hospital of Chongqing Medical University, National Clinical Research Center for Child Health and Disorders, Ministry of Education Key Laboratory of Child Development and Disorders, Chongqing, China; ^2^Chongqing Key Laboratory of Pediatrics, Chongqing Engineering Research Center of Stem Cell Therapy, Chongqing, China; ^3^Clinical Research Unit, Children’s Hospital of Shanghai Jiao Tong University, Shanghai, China; ^4^Institute of Pediatric Infection, Immunity, and Critical Care Medicine, Shanghai Jiao Tong University School of Medicine, Shanghai, China

**Keywords:** mesenchymal stem cells, exosome, extracellular vesicles, acute lung injury, COVID-19, sepsis, infectious diseases

## Abstract

Extracellular vesicles (EVs) are nano-sized membrane vesicles secreted by cells. EVs serve as a mediator for cell-to-cell communication by regulating the exchange of genetic materials and proteins between the donor and surrounding cells. Current studies have explored the therapeutic value of mesenchymal stem cells-derived EVs (MSC-EVs) for the treatment of infectious diseases extensively. MSC-EVs can eliminate the pathogen, regulate immunity, and repair tissue injury in contagious diseases through the secretion of antimicrobial factors, inhibiting the replication of pathogens and activating the phagocytic function of macrophages. MSC-EVs can also repair tissue damage associated with the infection by upregulating the levels of anti-inflammatory factors, downregulating the pro-inflammatory factors, and participating in the regulation of cellular biological behaviors. The purpose of this mini-review is to discuss in detail the various mechanisms of MSC-EV treatment for infectious diseases including respiratory infections, sepsis, and intestinal infections, as well as challenges for implementing MSC-EVs from bench to bedside.

## Introduction

Infectious diseases have been a significant cause of morbidity and mortality worldwide; respiratory infections and pneumonia are among the major causes of global death (Sharma et al., 2021b). With the increasing number of outbreaks of new infectious diseases and the lack of effective treatments, it is crucial to identify new therapeutic strategies to combat infections and restore infection-related organ and tissue damage.

Mesenchymal stem cells (MSCs) are among the most commonly employed cell types in tissue repair and homeostasis, which have become an attractive therapeutic option for treating infectious diseases and disease-related tissue injury (Kashte et al., 2018; Kotas and Matthay, 2018). The effects of MSCs include anti-inflammatory properties, immunomodulatory capabilities, and regeneration (Fu et al., 2019). The efficacy of MSCs is mainly coming from the paracrine effect mediated by secreted growth factors, cytokines, and extracellular vesicles (EVs) (Liang et al., 2014; Paliwal et al., 2018).

MSC-derived extracellular vesicles (MSC-EVs) are identified to be the main components responsible for the paracrine effect. They transfer functional molecules, such as messenger RNA (mRNA), microRNA (miRNA), lipid, and protein, into tissue-specific cells that request repair (Taverna et al., 2017). Compared with MSCs, MSC-EVs possess hypoimmunogenic properties, have low tumorigenesis, and are more stable (Trounson and McDonald, 2015). In this mini-review, we briefly summarize the function of exosomes and discuss their potential role in therapeutic regimens in infectious diseases, including respiratory infections, sepsis, and intestinal infections in recent years.

## Extracellular Vesicles From Mesenchymal Stem Cells

**FIGURE 1:**
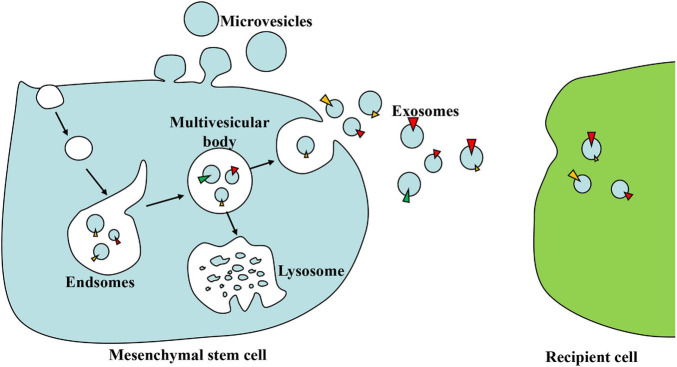
The biogenesis and action of exosomes. Early endosomes are formed by the endocytosis of the cell membrane and then develop into multivesicular bodies (MVBs) in a budding manner. MVBs either combine with lysosomes and digest their contents or be released as exosomes through exocytosis. Exosomes can deliver lipids, proteins, and nucleic acid to recipient cells when circulating in the extracellular space.

Almost all cells, including MSCs, can secrete EVs due to intracellular vesicle sorting (Kourembanas, 2015). EVs are nano-sized spherical bio-membrane structures, which were previously divided into three main categories based on their size and biosynthesis: smaller-sized exosomes (30–100 nm) from the endocytic pathway, medium-sized microvesicles (MVs) (100–1,000 nm) from the cell plasma membrane shedding, and larger-sized apoptotic bodies (1,000–5,000 nm) from the apoptosis (Raposo and Stoorvogel, 2013). The endocytosis of the cell membrane may form early endosomes, which then develop into late endosomes, namely, multivesicular bodies (MVBs). MVBs either combine with lysosomes or be released as exosomes through exocytosis (Joo et al., 2020). In terms of MVs, they can be secreted directly by budding from the plasma membrane (Abbaszadeh et al., 2020) (Figure 1).

Assigning an EV to a particular biogenesis pathway remains extraordinarily difficult because of the absence of specific surface markers for three EV categories and the overlap in their physical size (Carnino et al., 2021). Therefore, guidelines set by the International Society for Extracellular Vesicles (ISEV) suggest considering the use of operational terms for EV subtypes that are based on: (a) physical characteristics of EVs, such as size [“small EVs” (< 200 nm) and “medium/large EVs” (> 200 nm)] or density (low, middle, high, with each range defined); (b) biochemical composition (CD63^+^/CD81^+^-EVs, Annexin A5-stained EVs, etc.); or (c) descriptions of conditions or cell of origin (podocyte EVs, hypoxic EVs, large oncosomes, apoptotic bodies) (Théry et al., 2018).

Over 80% of researchers chose differential ultracentrifugation for EVs isolation (Tkach and Théry, 2016). Traditional identification ways for EVs usually involve nanoparticle tracking analysis (NTA) for size information, transmission electron microscope (TEM) for morphological details, and Western blotting for membrane protein makers (Théry et al., 2018). Kim et al. (2019) recently developed an atomic force microscope-infrared spectroscopy (AFM-IR) approach to probe the structural composition of a single EV. Their protocol involves incubating the EV sample on a suitable substrate and setting up the AFM-IR instrument, as well as collecting nano-IR spectra and nano-IR images. Recorded IR spectra for EVs showed characteristic peaks at specific wavenumbers; it is possible to determine the presence of DNA (1,050–1,290 cm^–1^), RNA (1,250–1,380 cm^–1^), proteins (1,500–1,700 cm^–1^), and phospholipids (1,000–1,250 cm^–1^, 1,730–1,750 cm^–1^, 2,800–3,000 cm^–1^) (Kim et al., 2019) that may contribute to the understanding of EV biology and the development of EV therapies. This method could improve the understanding of EV biology and the development of EV therapies.

EVs secreted from MSCs can deliver many functional molecules such as mRNA, miRNA, lipids, and protein into recipient cells (Yin et al., 2019). These biological components are considered stable and can modulate cell behaviors in recipient cells. EVs use specific receptors or membrane fusion to enter recipient cells. Once EVs are absorbed, the biomolecules of EVs can regulate gene expression, essential enzyme reactions, signal cascade pathways, or other mechanisms in recipient cells (Ranghino et al., 2017). Thus, MSC-EVs can promote tissue regeneration by reprogramming several pathophysiological pathways such as immunomodulation, proliferation, apoptosis, angiogenesis, and oxidative (Grange et al., 2019a, b).

## The Therapeutic Application of Mesenchymal Stem Cell-Extracellular Vesicles in Infectious Diseases

The function of EVs is mainly dependent on their source cells (Keshtkar et al., 2018). The therapeutic use of MSCs was reported in lung injury, sepsis, and necrotizing enterocolitis (NEC) caused by bacteria or viruses (Krasnodembskaya et al., 2010; Sung et al., 2016; Rodrigues et al., 2019). MSC-EVs have similar functions to their parental cells, such as antimicrobial effects, immunomodulation property, and damage tissue repairability. Compared with MSCs, MSC-EVs keep the biological function of MSCs and are more stable and less easy to tumorigenesis, making them a promising candidate for the treatment of infectious diseases (Thirabanjasak et al., 2010).

### For Respiratory Infection

Acute lung injury (ALI)/acute respiratory distress syndrome (ARDS) is a heterogeneous syndrome characterized by diffuse epithelial and endothelial damage and a robust inflammatory response (Thompson et al., 2017). The most common risk factors of ARDS are infectious pneumonia caused by bacteria and viruses (Muraca et al., 2020; Meyer et al., 2021). Respiratory infections take more than 1.5 million lives a year. The number of deaths and disabled people is devastating in epidemic and pandemic outbreaks, such as the severe acute respiratory syndrome (SARS) outbreak in 2002, H1N1 flu in 2009, Middle East respiratory syndrome (MERS) outbreak in 2012, and coronavirus disease 2019 (COVID-19) outbreak in 2020 (Sharma et al., 2021b).

Cell-based therapy with MSCs has been promising in ALI/ARDS in pre-clinical models for their immunomodulation and tissue repair properties (Laffey and Matthay, 2017). However, there were higher mean scores of Acute Physiology and Chronic Health Evaluation III (APACHE III) in models treated with MSCs than in those treated with placebo, but without difference of their 28-day mortality (Matthay et al., 2019). Since MSCs have limited engraftment and differentiation efficacy, high risk of tumorigenicity, and unstable ability (Eggenhofer et al., 2014), researchers paid more attention to MSC-EVs as a new candidate cell-free treatment for ALI/ARDS. Both other researchers and we demonstrated that intratracheal administration of MSC-EVs showed therapeutic effects in hyperoxia-induced lung injury, revealing that MSC-EVs could ameliorate impaired alveolarization in both short-term and long-term bronchopulmonary dysplasia (BPD) models and activate M2 macrophages (Porzionato et al., 2019, 2021; You et al., 2020). The anti-inflammatory and pro-regenerative properties of MSC-EVs are well established and have been exploited in a large number of studies (Phinney and Pittenger, 2017).

The application of MSC-EVs on ALI/ARDS and severe pneumonia has been investigated in some pre-clinical studies. MSC-EVs’ main effects on ALI/ARDS are reducing inflammation, promoting alveolar epithelial regeneration, and enhancing pulmonary endothelial repair (Shah et al., 2019). As a result, pro-inflammatory cytokine production was decreased, and alveolar fluid clearance was improved in ALI/ARDS models.

Two clinical trials are undergoing to determine the effects of MSC-EVs on COVID-19, a pandemic that lacks specific antiviral medicine. MSC-EVs will be administrated intravenously (NCT04798716) or by inhalation (NCT04276987). A prospective non-randomized open-label cohort study showed that allogeneic bone marrow MSC-derived exosomes (ExoFlo^TM^) could be safe and effective in severe COVID-19 patients, which could restore oxygenation, downregulate cytokine storm, and reconstitute immunity (Sengupta et al., 2020). However, it is premature to draw any conclusion based on a single study, and it should be emphasized that there are no approved MSC-EV therapies for COVID-19 to date. The specific and scientific rationale for administering MSC-EV treatment in COVID-19 patients needs to be better understood and justified (Börger et al., 2020). In the meantime, the prevention and control of urgent COVID-19 should make efforts to test existing approved vaccines, antiviral therapeutics, and monoclonal antibodies (Sharma et al., 2021a).

miRNA, protein, mRNA, and mitochondria in MSC-EVs play vital roles in modulating immune responses and repairing lung damage of ALI/ARDS. miR-21-5p plays an essential role in alleviating ALI by reducing pro-inflammatory cytokine secretion and enhancing M2 polarization (Li et al., 2019). MSC-EVs are reported to ameliorate ALI *via* transferring miR-27a-3p to alveolar macrophages inhibiting NF-κB expression and inducing M2 polarization (Wang et al., 2020). MiR-145 mediated the antimicrobial effect of MSC-EV by suppressing the expression of multidrug resistance-associated protein 1 (MRP1) and increasing the levels of leukotriene B_4_ (LTB_4_) (Hao et al., 2019), a chemoattractant for immune cells including T cells, macrophages, and neutrophils, with the role of facilitating pathogen elimination (Saeki and Yokomizo, 2017).

EVs from interferon (IFN)-γ-primed MSCs more effectively attenuated *Escherichia coli*-induced lung injury *via* enhancing phagocytosis and killing of bacteria in macrophage (Varkouhi et al., 2019). MSC-EVs decreased the lipopolysaccharide (LPS)-induced permeability of microvascular endothelial cells partly through the presence of hepatocyte growth factor (HGF) (Wang et al., 2017). The expression of keratinocyte growth factor (KGF) (Zhu et al., 2014) and angiopoietin-1 (Ang1) (Tang et al., 2017) mRNA enclosed in EVs partly mediated the anti-inflammatory effects on *E. coli* endotoxin-induced ALI in mice models. The effectiveness of MSC-EVs has also been demonstrated in large animals and found that EVs from swine bone marrow-derived MSCs had anti-influenza and anti-inflammatory effects in influenza virus-induced pig ALI (Khatri et al., 2018).

### For Sepsis

Sepsis is a systemic inflammatory response to infection that leads to multiple organ dysfunction, and one out of four sepsis patients died during their hospital stay (Iskander et al., 2013; Fleischmann-Struzek et al., 2020). Sepsis is caused by the accumulation of various pro-inflammatory factors in the process of inflammatory response and immune dysfunction (Prescott and Angus, 2018). Even with the continuous development of intensive care and advances in the antibiotic application, the mortality of sepsis in intensive care units remains high (Angus and van der Poll, 2013). Therefore, a new therapy is urgent to improve the clinical outcomes.

Patients with sepsis had severe immunosuppression, leading to macrophage dysfunction and poor wound healing (Davis et al., 2019). Therefore, the new therapy strategy could be related to the immunoregulation of macrophages. Several studies have proven that MSC-EVs can improve the outcomes of sepsis in animal models. MiRNAs in MSC-EVs have been considered as a critical substance to exert efficacy in sepsis. For example, miRNA-146a was found to be strongly upregulated in MSC-EVs primed with interleukin-1β (IL-1β), which could more effectively induce M2 polarization by modulating IRAK1, TRAF6, and IRF5 signaling (Song et al., 2017). MiR-21 in MSC-EVs was abundantly upregulated in IL-1β-stimulated MSCs, which induced M2 polarization of macrophages *in vitro* and *in vivo* sepsis by inhibiting the effects of PDCD4, which can participate in multiple cellular biological behaviors, including apoptosis and transcription (Yao et al., 2021). Both studies supported that pretreated MSCs with pro-inflammatory cytokines could enhance their immunomodulatory function of MSCs. The exosomal miR-223 was reported to contribute to MSC-mediated cardioprotection in sepsis by downregulation of Sema3A and STAT3 (Wang et al., 2015).

### For Intestinal Infection

The balance between beneficial and harmful bacteria plays an important role in neonatal intestinal health (Rhoads et al., 2018). Bacterial infection is one of the most significant risk factors in NEC pathogenesis, a life-threatening disease in premature infants, with mortality as high as 30% (Neu and Walker, 2011; Markel et al., 2020). Full-thickness destruction of the intestine is the character of NEC, and inflammatory response is increased in infants affected by this disease, leading to intestinal perforation, peritonitis, bacterial invasion of the bloodstream, and systemic infection (Neu, 2014; Neu and Pammi, 2018). Survivors are faced with severe sequelae, including short gut syndrome and neurodevelopmental retardation (Neu, 2014). Despite decades of research on the pathophysiology of NEC, the treatment remains inadequate and supportive and desired a novel preventive and therapeutic intervention.

MSCs have great potential in NEC treatment, decreasing NEC incidence in rat models (Augustine et al., 2017; Thébaud, 2019). EVs from MSCs carry important biological components and can be utilized in disease prevention and treatment (Baglio et al., 2015). EVs from bone marrow-derived MSCs, heparin-binding EGF-like growth factor (HB-EGF) primed MSCs, and human umbilical cord MSCs have been reported to protect the integrity of the intestinal barrier and reduce the severity and incidence of NEC in an experimental model (Rager et al., 2016; McCulloh et al., 2018). Both miR-34 and miR-29 improved the intestinal epithelial barrier through the Snail/Claudins signaling pathway (Li et al., 2020). MiR-200b in heme oxygenase-1 (HO-1)-modified bone marrow MSCs-derived EVs was reported to target high mobility group box 3 (HMGB3) gene in intestinal epithelial cells to alleviate its inflammatory response (Sun et al., 2020b).

### For Other Infectious Diseases

Qian et al. (2016) revealed that miRNAs, especially let-7f, miR-145, miR-199, and miR-221 from MSC-EVs, inhibited viral replication in hepatitis C virus (HCV)-treated cells. Hepatocyte injury model caused by D-galactosamine (D-GaIN) and LPS could be ameliorated by MSC-EVs through inducing autophagy and inhibiting apoptosis (Zhao et al., 2019). In addition, MSC-EVs had therapeutic effects on coxsackievirus (CVB3)-induced myocarditis in the mice model, which can shrink the production of pro-inflammatory cytokines and improve cardiac function *via* activating the AMPK/mTOR-mediated autophagy flux pathway to attenuate apoptosis (Gu et al., 2020).

## Conclusion

MSC-EVs had outstanding prospects in treating infectious diseases, such as respiratory infections, sepsis, and intestinal infections. The therapeutic mechanisms included direct antimicrobial effects, immunomodulation, and tissue repair. MSC-EVs exert their effect through the transfer of mRNAs, miRNAs, and proteins (Table 1). MiRNA containing EV may be a new target for the development of new therapeutic drugs. The use of MSC-EVs has several benefits, namely, (a) small vesicles, readily circulating and penetrating biological barriers, like blood–brain; (b) low tumorigenesis; and (c) stable properties, MSC-EVs may achieve a higher “dose” than MSCs due to the poor viability and considerable death of engrafted MSCs in target tissues (Barbash et al., 2003). Importantly, EVs can maintain high activities at low temperatures. All the profits make MSC-EVs a promising agent in infectious diseases.

**TABLE 1:** The related exosomal cargo and mechanisms of mesenchymal stem cell-derived extracellular vesicles treatment in infectious diseases.

**Related exosomal cargo**	**Disease model**	**Exosome source**	**MSC-EV isolation**	**Experimental outcome and related mechanism**
miR-27a-3p (Wang et al., 2020)	LPS-induced ALI in mouse	hADMSCs	UC	Elevated miR-27-3a levels in alveolar macrophages, induced M2 polarization, and decreased alveolar macrophage expression of NF-κB
miR-145 (Hao et al., 2019)	*E. coli*-induced ALI in mouse	hBMSCs	UC	Suppressed MRP1 activity through transfer of miR-145, thereby resulting in enhanced LTB_4_ production and antimicrobial activity through LTB_4_/BLT1 signaling
Unknown (Varkouhi et al., 2019)	*E. coli*-induced ALI in rat	IFN-γ-primed hUCMSCs	UC	Enhanced macrophage phagocytosis and killing of *E. coli*
HGF (Wang et al., 2017)	*In vitro* LPS treatment of endothelial cells	mBMSCs	UC	Increased the expression of VE-cadherin and occluding, decreased endothelial apoptosis, induced endothelial cell proliferation
KGF (Zhu et al., 2014), Ang-1 (Tang et al., 2017)	*E. coli*/LPS-induced ALI in mouse	hBMSCs	UC	Demonstrated a reduction in pulmonary edema, lung protein permeability, and inflammation
RNAs (Khatri et al., 2018)	Influenza virus-induced ALI in pig	sBMSCs	UC	Reduced virus shedding in the nasal swabs, influenza virus replication, and pro-inflammatory cytokines in the lungs
miR-146a (Song et al., 2017), miR-21 (Yao et al., 2021)	CLP-induced sepsis in mouse	IL-1β primed hUCMSCs	UC	Exosomal miR-146a/miR-21 was transferred to macrophages, resulted in M2 polarization by modulating IRAK1, TRAF6, and IRF 5 signaling, or inhibited the effects of PDCD4.
miR-223 (Wang et al., 2015)	CLP-induced sepsis in mouse	mBMSCs	UC	Exosomal miR-223 was transferred to cardiomyocytes, inhibited the expression of Sema3A and Stat3, and reduced inflammation and cell death.
Unknown (Rager et al., 2016; McCulloh et al., 2018)	Premature and hypercaloric feeds-induced NEC in rat	rAFMSCs, rBMSCs, and mBMSCs	UC	Reduced the incidence and severity of experimental NEC and protected the intestines from NEC
miR-200b (Sun et al., 2020b)	*In vitro* TNF-α treatment of endothelial cells	HO-1-modified rBMSCs	Exosome separation kits	Targeted HMGB3 in intestinal epithelial cells to alleviate inflammatory injury
Let-7f, miR-145, miR-199a, and miR-221 (Qian et al., 2016)	*In vitro* HCV treatment of human hepatoma-7 cells	hBMSCs	UC	Suppression of HCV RNA replication, combined with INF-α or telaprevir, enhanced their anti-HCV ability
Unknown (Gu et al., 2020)	*In vitro* D-GaIN/LPS treatment of hepatocytes	BMSCs	UC	Decreased the expression levels of the pro-apoptotic proteins Bax and cleaved caspase-3, upregulated the anti-apoptotic protein Bcl-2, reduced hepatocyte apoptosis
Unknown (Sun et al., 2020a)	CVB3-induced myocarditis in mouse	hBMSCs	UC	Activated AMPK/mTOR-mediated autophagy flux pathway to attenuate cardiomyocyte apoptosis

*MSC-EV, mesenchymal stem cell-derived extracellular vesicle; hADMSCs, human adipose-derived MSCs; hBMSCs, human bone marrow-derived MSCs; hUCMSCs, human umbilical cord-derived MSCs; mBMSCs, mouse bone marrow-derived MSCs; sBMSCs, swine bone marrow-derived MSCs; rAFMSCs, rat amniotic fluid-derived MSCs; rBMSCs, rat bone marrow-derived MSCs; HCV, hepatitis C virus; HGF, hepatocyte growth factor; KGF, keratinocyte growth factor; Ang-1, angiopoietin-1; LPS, lipopolysaccharide; ALI, acute lung injury; E. coli, Escherichia coli; CLP, cecal ligation and puncture; NEC, necrotizing enterocolitis; D-GaIN, D-galactosamine hydrochloride; CVB3, coxsackievirus B3; HO-1, heme oxygenase-1; IL-1β, interleukin-1β; TNF-α, tumor necrosis factor-α; UC, ultracentrifugation; NF-κB, nuclear factor kappa B subunit 1; MRP1, multidrug resistance-associated protein 1; LTB_4_, leukotriene (LT) B_4;_ HMGB3, high mobility group box 3.*

Despite the promising progress that has been made in the treatment of MSC-EVs on infectious diseases, several challenges are faced by the field in clinical translation: (a) there is wide variability of MSC-EVs preparations in the whole process (Börger et al., 2020), such as the different productions of cell sources, purification, and identification of the final product. Careful consideration of the optimal purity and rational clinical trial design of MSC-EVs is necessary to advance large-scale clinical trials (Muraca et al., 2018). Furthermore, lacking standardized quality parameters caused discrepancies and controversies about the biology and function of MSC-EVs. Members of four societies (SOCRATES, ISCT, ISEV, and ISBT) identified potential metrics of MSC-EVs to facilitate data sharing and comparison of MSC-EVs among different studies, including biological activity, vesicle integrity, the concentration of membrane lipid vesicles, the ratio of specific lipids, the ratio of membrane lipids to protein, and the ratio of MSC to non-MSC surface antigens (Witwer et al., 2019). Each metric needs to be quantified and validated in further studies. (b) How to determine reproducible and robust parameters to predict the therapeutic potency of MSC-EVs is unsolved. The therapeutic efficacy of MSC-EVs depends not only on the cell, such as the cell source and status of MSCs, delivery dose and route (Sun et al., 2020a), and half-life and *in vivo* biodistribution of MSC-EVs, but also on the disease condition, such as the disease microenvironment and the time window for intervention. (c) MSC-EVs from different sources have been reported to be efficacious in various kinds of infectious diseases; the therapeutic mechanism may be different and specific for each source and disease condition. To better understand the therapeutic activity, the mode of action needs to be studied further, trying to find out the key components in MSC-EVs, target cells in injured tissues, and the involved molecular signaling cascade.

## Author Contributions

LZ: conceptualization and review. ZF: supervision. JY: writing and editing. All authors contributed to the article and approved the submitted version.

## Conflict of Interest

The authors declare that the research was conducted in the absence of any commercial or financial relationships that could be construed as a potential conflict of interest.

## Publisher’s Note

All claims expressed in this article are solely those of the authors and do not necessarily represent those of their affiliated organizations, or those of the publisher, the editors and the reviewers. Any product that may be evaluated in this article, or claim that may be made by its manufacturer, is not guaranteed or endorsed by the publisher.

## References

[B1] Abbaszadeh H. Ghorbani F. Derakhshani M. Movassaghpour A. Yousefi M. (2020). Human umbilical cord mesenchymal stem cell-derived extracellular vesicles: a novel therapeutic paradigm. *J. Cell. Physiol.* 235 706–717. 10.1002/jcp.29004 31254289

[B2] Angus D. C. van der Poll T. (2013). Severe sepsis and septic shock. *N. Engl. J. Med.* 369 840–851. 23984731 10.1056/NEJMra1208623

[B3] Augustine S. Avey M. T. Harrison B. Locke T. Ghannad M. Moher D. (2017). Mesenchymal stromal cell therapy in bronchopulmonary dysplasia: systematic review and meta-analysis of preclinical studies. *Stem Cells Transl. Med.* 6 2079–2093. 10.1002/sctm.17-0126 29045045 PMC5702524

[B4] Baglio S. R. Rooijers K. Koppers-Lalic D. Verweij F. J. Pérez Lanzón M. Zini N. (2015). Human bone marrow- and adipose-mesenchymal stem cells secrete exosomes enriched in distinctive miRNA and tRNA species. *Stem Cell Res. Ther.* 6:127. 26129847 10.1186/s13287-015-0116-z PMC4529699

[B5] Barbash I. M. Chouraqui P. Baron J. Feinberg M. S. Etzion S. Tessone A. (2003). Systemic delivery of bone marrow-derived mesenchymal stem cells to the infarcted myocardium: feasibility, cell migration, and body distribution. *Circulation* 108 863–868. 10.1161/01.cir.0000084828.50310.6a 12900340

[B6] Börger V. Weiss D. J. Anderson J. D. Borràs F. E. Bussolati B. Carter D. R. F. (2020). International society for extracellular vesicles and international society for cell and gene therapy statement on extracellular vesicles from mesenchymal stromal cells and other cells: considerations for potential therapeutic agents to suppress coronavirus disease-19. *Cytotherapy* 22 482–485. 10.1016/j.jcyt.2020.05.002 32425691 PMC7229942

[B7] Carnino J. M. Hao Kwok Z. Jin Y. (2021). Extracellular vesicles: a novel opportunity for precision medicine in respiratory diseases. *Front. Med.* 8:661679. 10.3389/fmed.2021.661679 34368181 PMC8342920

[B8] Davis F. M. Schaller M. A. Dendekker A. Joshi A. D. Kimball A. S. Evanoff H. (2019). Sepsis induces prolonged epigenetic modifications in bone marrow and peripheral macrophages impairing inflammation and wound healing. *Arterioscler. Thromb. Vasc. Biol.* 39 2353–2366. 10.1161/atvbaha.119.312754 31644352 PMC6818743

[B9] Eggenhofer E. Luk F. Dahlke M. H. Hoogduijn M. J. (2014). The life and fate of mesenchymal stem cells. *Front. Immunol.* 5:148. 10.3389/fimmu.2014.00148 24904568 PMC4032901

[B10] Fleischmann-Struzek C. Mellhammar L. Rose N. Cassini A. Rudd K. E. Schlattmann P. (2020). Incidence and mortality of hospital- and ICU-treated sepsis: results from an updated and expanded systematic review and meta-analysis. *Intensive Care Med.* 46 1552–1562. 10.1007/s00134-020-06151-x 32572531 PMC7381468

[B11] Fu X. Liu G. Halim A. Ju Y. Luo Q. Song A. G. (2019). Mesenchymal stem cell migration and tissue repair. *Cells* 8:784. 10.3390/cells8080784 31357692 PMC6721499

[B12] Grange C. Skovronova R. Marabese F. Bussolati B. (2019a). Stem cell-derived extracellular vesicles and kidney regeneration. *Cells* 8:1240. 10.3390/cells8101240 31614642 PMC6830104

[B13] Grange C. Tritta S. Tapparo M. Cedrino M. Tetta C. Camussi G. (2019b). Stem cell-derived extracellular vesicles inhibit and revert fibrosis progression in a mouse model of diabetic nephropathy. *Sci. Rep.* 9:4468. 30872726 10.1038/s41598-019-41100-9 PMC6418239

[B14] Gu X. Li Y. Chen K. Wang X. Wang Z. Lian H. (2020). Exosomes derived from umbilical cord mesenchymal stem cells alleviate viral myocarditis through activating AMPK/mTOR-mediated autophagy flux pathway. *J. Cell. Mol. Med.* 24 7515–7530. 10.1111/jcmm.15378 32424968 PMC7339183

[B15] Hao Q. Gudapati V. Monsel A. Park J. H. Hu S. Kato H. (2019). Mesenchymal stem cell-derived extracellular vesicles decrease lung injury in mice. *J. Immunol.* 203 1961–1972. 31451675 10.4049/jimmunol.1801534 PMC6760999

[B16] Iskander K. N. Osuchowski M. F. Stearns-Kurosawa D. J. Kurosawa S. Stepien D. Valentine C. (2013). Sepsis: multiple abnormalities, heterogeneous responses, and evolving understanding. *Physiol. Rev.* 93 1247–1288. 10.1152/physrev.00037.2012 23899564 PMC3962548

[B17] Joo H. S. Suh J. H. Lee H. J. Bang E. S. Lee J. M. (2020). Current knowledge and future perspectives on mesenchymal stem cell-derived exosomes as a new therapeutic agent. *Int. J. Mol. Sci.* 21:727. 10.3390/ijms21030727 31979113 PMC7036914

[B18] Kashte S. Maras J. S. Kadam S. (2018). Bioinspired engineering for liver tissue regeneration and development of bioartificial liver: a review. *Crit. Rev. Biomed. Eng.* 46 413–427. 10.1615/critrevbiomedeng.2018028276 30806261

[B19] Keshtkar S. Azarpira N. Ghahremani M. H. (2018). Mesenchymal stem cell-derived extracellular vesicles: novel frontiers in regenerative medicine. *Stem Cell Res. Ther.* 9:63. 29523213 10.1186/s13287-018-0791-7 PMC5845209

[B20] Khatri M. Richardson L. A. Meulia T. (2018). Mesenchymal stem cell-derived extracellular vesicles attenuate influenza virus-induced acute lung injury in a pig model. *Stem Cell Res. Ther.* 9:17. 29378639 10.1186/s13287-018-0774-8 PMC5789598

[B21] Kim S. Y. Khanal D. Kalionis B. Chrzanowski W. (2019). High-fidelity probing of the structure and heterogeneity of extracellular vesicles by resonance-enhanced atomic force microscopy infrared spectroscopy. *Nat. Protoc.* 14 576–593. 10.1038/s41596-018-0109-3 30651586

[B22] Kotas M. E. Matthay M. A. (2018). Mesenchymal stromal cells and macrophages in sepsis: new insights. *Eur. Respir. J.* 51:1800510. 10.1183/13993003.00510-2018 29700107

[B23] Kourembanas S. (2015). Exosomes: vehicles of intercellular signaling, biomarkers, and vectors of cell therapy. *Annu. Rev. Physiol.* 77 13–27. 10.1146/annurev-physiol-021014-071641 25293529

[B24] Krasnodembskaya A. Song Y. Fang X. Gupta N. Serikov V. Lee J. W. (2010). Antibacterial effect of human mesenchymal stem cells is mediated in part from secretion of the antimicrobial peptide LL-37. *Stem Cells* 28 2229–2238. 10.1002/stem.544 20945332 PMC3293245

[B25] Laffey J. G. Matthay M. A. (2017). Fifty years of research in ARDS. Cell-based therapy for acute respiratory distress syndrome. biology and potential therapeutic value. *Am. J. Respir. Crit. Care Med.* 196 266–273. 28306336 10.1164/rccm.201701-0107CP PMC5549868

[B26] Li J. W. Wei L. Han Z. Chen Z. (2019). Mesenchymal stromal cells-derived exosomes alleviate ischemia/reperfusion injury in mouse lung by transporting anti-apoptotic miR-21-5p. *Eur. J. Pharmacol.* 852 68–76. 10.1016/j.ejphar.2019.01.022 30682335

[B27] Li Y. Y. Xu Q. W. Xu P. Y. Li W. M. (2020). MSC-derived exosomal miR-34a/c-5p and miR-29b-3p improve intestinal barrier function by targeting the Snail/Claudins signaling pathway. *Life Sci.* 257:118017. 10.1016/j.lfs.2020.118017 32603821

[B28] Liang X. Ding Y. Zhang Y. Tse H. F. Lian Q. (2014). Paracrine mechanisms of mesenchymal stem cell-based therapy: current status and perspectives. *Cell Transplant.* 23 1045–1059. 10.3727/096368913x667709 23676629

[B29] Markel T. A. Martin C. A. Chaaban H. Canvasser J. Tanner H. Denchik H. (2020). New directions in necrotizing enterocolitis with early-stage investigators. *Pediatr. Res.* 88 35–40. 10.1038/s41390-020-1078-0 32855511 PMC7934965

[B30] Matthay M. A. Calfee C. S. Zhuo H. Thompson B. T. Wilson J. G. Levitt J. E. (2019). Treatment with allogeneic mesenchymal stromal cells for moderate to severe acute respiratory distress syndrome (START study): a randomised phase 2a safety trial. *Lancet Respir. Med.* 7 154–162. 10.1016/s2213-2600(18)30418-1 30455077 PMC7597675

[B31] McCulloh C. J. Olson J. K. Wang Y. Zhou Y. Tengberg N. H. Deshpande S. (2018). Treatment of experimental necrotizing enterocolitis with stem cell-derived exosomes. *J. Pediatr. Surg.* 53 1215–1220. 10.1016/j.jpedsurg.2018.02.086 29661576 PMC5994352

[B32] Meyer N. J. Gattinoni L. Calfee C. S. (2021). Acute respiratory distress syndrome. *Lancet* 398 622–637. 34217425 10.1016/S0140-6736(21)00439-6 PMC8248927

[B33] Muraca M. Pessina A. Pozzobon M. Dominici M. Galderisi U. Lazzari L. (2020). Mesenchymal stromal cells and their secreted extracellular vesicles as therapeutic tools for COVID-19 pneumonia? *J. Control. Release* 325 135–140. 10.1016/j.jconrel.2020.06.036 32622963 PMC7332437

[B34] Muraca M. Zaramella P. Porzionato A. Baraldi E. (2018). Exosome treatment of bronchopulmonary dysplasia: how pure should your exosome preparation be? *Am. J. Respir. Crit. Care Med.* 197 969–970. 10.1164/rccm.201709-1851le 29160724

[B35] Neu J. (2014). Necrotizing enterocolitis: the mystery goes on. *Neonatology* 106 289–295. 10.1159/000365130 25171544

[B36] Neu J. Pammi M. (2018). Necrotizing enterocolitis: the intestinal microbiome, metabolome and inflammatory mediators. *Semin. Fetal Neonatal Med.* 23 400–405. 10.1016/j.siny.2018.08.001 30172660

[B37] Neu J. Walker W. A. (2011). Necrotizing enterocolitis. *N. Engl. J. Med.* 364 255–264. 21247316 10.1056/NEJMra1005408 PMC3628622

[B38] Paliwal S. Chaudhuri R. Agrawal A. Mohanty S. (2018). Regenerative abilities of mesenchymal stem cells through mitochondrial transfer. *J. Biomed. Sci.* 25:31. 29602309 10.1186/s12929-018-0429-1 PMC5877369

[B39] Phinney D. G. Pittenger M. F. (2017). Concise review: MSC-derived exosomes for cell-free therapy. *Stem Cells* 35 851–858. 10.1002/stem.2575 28294454

[B40] Porzionato A. Zaramella P. Dedja A. Guidolin D. Bonadies L. Macchi V. (2021). Intratracheal administration of mesenchymal stem cell-derived extracellular vesicles reduces lung injuries in a chronic rat model of bronchopulmonary dysplasia. *Am. J. Physiol. Lung Cell. Mol. Physiol.* 320 L688–L704. 33502939 10.1152/ajplung.00148.2020

[B41] Porzionato A. Zaramella P. Dedja A. Guidolin D. Van Wemmel K. Macchi V. (2019). Intratracheal administration of clinical-grade mesenchymal stem cell-derived extracellular vesicles reduces lung injury in a rat model of bronchopulmonary dysplasia. *Am. J. Physiol. Lung Cell. Mol. Physiol.* 316 L6–L19. 30284924 10.1152/ajplung.00109.2018

[B42] Prescott H. C. Angus D. C. (2018). Enhancing recovery from sepsis: a review. *JAMA* 319 62–75. 10.1001/jama.2017.17687 29297082 PMC5839473

[B43] Qian X. Xu C. Fang S. Zhao P. Wang Y. Liu H. (2016). Exosomal MicroRNAs derived from umbilical mesenchymal stem cells inhibit hepatitis C virus infection. *Stem Cells Transl. Med.* 5 1190–1203. 10.5966/sctm.2015-0348 27496568 PMC4996444

[B44] Rager T. M. Olson J. K. Zhou Y. Wang Y. Besner G. E. (2016). Exosomes secreted from bone marrow-derived mesenchymal stem cells protect the intestines from experimental necrotizing enterocolitis. *J. Pediatr. Surg.* 51 942–947. 10.1016/j.jpedsurg.2016.02.061 27015901 PMC4921266

[B45] Ranghino A. Bruno S. Bussolati B. Moggio A. Dimuccio V. Tapparo M. (2017). The effects of glomerular and tubular renal progenitors and derived extracellular vesicles on recovery from acute kidney injury. *Stem Cell Res. Ther.* 8:24. 28173878 10.1186/s13287-017-0478-5 PMC5297206

[B46] Raposo G. Stoorvogel W. (2013). Extracellular vesicles: exosomes, microvesicles, and friends. *J. Cell Biol.* 200 373–383. 23420871 10.1083/jcb.201211138 PMC3575529

[B47] Rhoads J. M. Collins J. Fatheree N. Y. Hashmi S. S. Taylor C. M. Luo M. (2018). Infant colic represents gut inflammation and dysbiosis. *J. Pediatr.* 203 55–61.e53. 30177353 10.1016/j.jpeds.2018.07.042 PMC6669027

[B48] Rodrigues E. S. de Macedo M. D. Orellana M. D. Takayanagui O. M. Palma P. V. B. Pinto M. T. (2019). Short communication: human bone marrow stromal cells exhibit immunosuppressive effects on human T lymphotropic virus Type 1 T lymphocyte from infected individuals. *AIDS Res. Hum. Retroviruses* 35 164–168. 10.1089/aid.2018.0066 30351194

[B49] Saeki K. Yokomizo T. (2017). Identification, signaling, and functions of LTB(4) receptors. *Semin. Immunol.* 33 30–36. 10.1016/j.smim.2017.07.010 29042026

[B50] Sengupta V. Sengupta S. Lazo A. Woods P. Nolan A. Bremer N. (2020). Exosomes derived from bone marrow mesenchymal stem cells as treatment for severe COVID-19. *Stem Cells Dev.* 29 747–754. 10.1089/scd.2020.0080 32380908 PMC7310206

[B51] Shah T. G. Predescu D. Predescu S. (2019). Mesenchymal stem cells-derived extracellular vesicles in acute respiratory distress syndrome: a review of current literature and potential future treatment options. *Clin. Transl. Med.* 8:25. 31512000 10.1186/s40169-019-0242-9 PMC6739436

[B52] Sharma A. Chakraborty A. Jaganathan B. G. (2021b). Review of the potential of mesenchymal stem cells for the treatment of infectious diseases. *World J. Stem Cells* 13 568–593. 10.4252/wjsc.v13.i6.568 34249228 PMC8246252

[B53] Sharma A. Ahmad Farouk I. Lal S. K. (2021a). COVID-19: a review on the novel coronavirus disease evolution, transmission, detection, control and prevention. *Viruses* 13:202. 10.3390/v13020202 33572857 PMC7911532

[B54] Song Y. Dou H. Li X. Zhao X. Li Y. Liu D. (2017). Exosomal miR-146a contributes to the enhanced therapeutic efficacy of interleukin-1β-primed mesenchymal stem cells against sepsis. *Stem Cells* 35 1208–1221. 28090688 10.1002/stem.2564

[B55] Sun D. Cao H. Yang L. Lin L. Hou B. Zheng W. (2020b). MiR-200b in heme oxygenase-1-modified bone marrow mesenchymal stem cell-derived exosomes alleviates inflammatory injury of intestinal epithelial cells by targeting high mobility group box 3. *Cell Death Dis.* 11:480. 32587254 10.1038/s41419-020-2685-8 PMC7316799

[B56] Sun C. Wang L. Wang H. Huang T. Yao W. Li J. (2020a). Single-cell RNA-seq highlights heterogeneity in human primary Wharton’s jelly mesenchymal stem/stromal cells cultured *in vitro*. *Stem Cell Res. Ther.* 11:149. 32252818 10.1186/s13287-020-01660-4 PMC7132901

[B57] Sung D. K. Chang Y. S. Sung S. I. Yoo H. S. Ahn S. Y. Park W. S. (2016). Antibacterial effect of mesenchymal stem cells against *Escherichia coli* is mediated by secretion of beta- defensin- 2 via toll- like receptor 4 signalling. *Cell Microbiol.* 18 424–436. 10.1111/cmi.12522 26350435 PMC5057339

[B58] Tang X. D. Shi L. Monsel A. Li X. Y. Zhu H. L. Zhu Y. G. (2017). Mesenchymal stem cell microvesicles attenuate acute lung injury in mice partly mediated by Ang-1 mRNA. *Stem Cells* 35 1849–1859. 10.1002/stem.2619 28376568

[B59] Taverna S. Pucci M. Alessandro R. (2017). Extracellular vesicles: small bricks for tissue repair/regeneration. *Ann. Transl. Med.* 5:83. 28275628 10.21037/atm.2017.01.53 PMC5337202

[B60] Thébaud B. (2019). Stem cells for extreme prematurity. *Am. J. Perinatol.* 36 S68–S73. 31238363 10.1055/s-0039-1691774

[B61] Théry C. Witwer K. W. Aikawa E. Alcaraz M. J. Anderson J. D. Andriantsitohaina R. (2018). Minimal information for studies of extracellular vesicles 2018 (MISEV2018): a position statement of the International Society for Extracellular Vesicles and update of the MISEV2014 guidelines. *J. Extracell. Vesicles* 7:1535750. 30637094 10.1080/20013078.2018.1535750 PMC6322352

[B62] Thirabanjasak D. Tantiwongse K. Thorner P. S. (2010). Angiomyeloproliferative lesions following autologous stem cell therapy. *J. Am. Soc. Nephrol.* 21 1218–1222. 10.1681/asn.2009111156 20558536 PMC3152236

[B63] Thompson B. T. Chambers R. C. Liu K. D. (2017). Acute respiratory distress syndrome. *N. Engl. J. Med.* 377 562–572. 28792873 10.1056/NEJMra1608077

[B64] Tkach M. Théry C. (2016). Communication by extracellular vesicles: where we are and where we need to go. *Cell* 164 1226–1232. 26967288 10.1016/j.cell.2016.01.043

[B65] Trounson A. McDonald C. (2015). Stem cell therapies in clinical trials: progress and challenges. *Cell Stem Cell* 17 11–22. 26140604 10.1016/j.stem.2015.06.007

[B66] Varkouhi A. K. Jerkic M. Ormesher L. Gagnon S. Goyal S. Rabani R. (2019). Extracellular vesicles from interferon-γ-primed human umbilical cord mesenchymal stromal cells reduce *Escherichia coli*-induced acute lung injury in rats. *Anesthesiology* 130 778–790. 10.1097/aln.0000000000002655 30870158

[B67] Wang H. Zheng R. Chen Q. Shao J. Yu J. Hu S. (2017). Mesenchymal stem cells microvesicles stabilize endothelial barrier function partly mediated by hepatocyte growth factor (HGF). *Stem Cell Res. Ther.* 8:211. 28969681 10.1186/s13287-017-0662-7 PMC5623961

[B68] Wang J. Huang R. Xu Q. Zheng G. Qiu G. Ge M. (2020). Mesenchymal stem cell-derived extracellular vesicles alleviate acute lung injury via transfer of miR-27a-3p. *Crit. Care Med.* 48 e599–e610. 32317602 10.1097/CCM.0000000000004315

[B69] Wang X. Gu H. Qin D. Yang L. Huang W. Essandoh K. (2015). Exosomal miR-223 contributes to mesenchymal stem cell-elicited cardioprotection in polymicrobial sepsis. *Sci. Rep.* 5:13721. 26348153 10.1038/srep13721 PMC4562230

[B70] Witwer K. W. Van Balkom B. W. M. Bruno S. Choo A. Dominici M. Gimona M. (2019). Defining mesenchymal stromal cell (MSC)-derived small extracellular vesicles for therapeutic applications. *J. Extracell. Vesicles* 8:1609206. 10.1080/20013078.2019.1609206 31069028 PMC6493293

[B71] Yao M. Cui B. Zhang W. Ma W. Zhao G. Xing L. (2021). Exosomal miR-21 secreted by IL-1β-primed-mesenchymal stem cells induces macrophage M2 polarization and ameliorates sepsis. *Life Sci.* 264:118658. 10.1016/j.lfs.2020.118658 33115604

[B72] Yin K. Wang S. Zhao R. C. (2019). Exosomes from mesenchymal stem/stromal cells: a new therapeutic paradigm. *Biomark. Res.* 7:8. 10.1002/9781118907474.ch2 30992990 PMC6450000

[B73] You J. Zhou O. Liu J. Zou W. Zhang L. Tian D. (2020). Human umbilical cord mesenchymal stem cell-derived small extracellular vesicles alleviate lung injury in rat model of bronchopulmonary dysplasia by affecting cell survival and angiogenesis. *Stem Cells Dev.* 29 1520–1532. 10.1089/scd.2020.0156 33040709

[B74] Zhao S. Liu Y. Pu Z. (2019). Bone marrow mesenchymal stem cell-derived exosomes attenuate D-GaIN/LPS-induced hepatocyte apoptosis by activating autophagy *in vitro*. *Drug Des. Devel. Ther.* 13 2887–2897. 10.2147/dddt.s220190 31695322 PMC6707369

[B75] Zhu Y. G. Feng X. M. Abbott J. Fang X. H. Hao Q. Monsel A. (2014). Human mesenchymal stem cell microvesicles for treatment of *Escherichia coli* endotoxin-induced acute lung injury in mice. *Stem Cells* 32 116–125. 10.1002/stem.1504 23939814 PMC3947321

